# Outcomes after the surgery for acquired nonaccommodative esotropia

**DOI:** 10.1186/s12886-017-0527-y

**Published:** 2017-07-24

**Authors:** Eunbi Kim, Dong Gyu Choi

**Affiliations:** Department of Ophthalmology, Hallym University College of Medicine, Kangnam Sacred Heart Hospital, Shingil-ro 1, Youngdeungpo-gu, Seoul, 07441 South Korea

**Keywords:** Acquired nonaccommodative esotropia, Surgical outcome, Reoperation, Influencing factor

## Abstract

**Background:**

To analyze the surgical outcomes for patients diagnosed with acquired nonaccommodative esotropia (ANAET).

**Methods:**

In this retrospective study, the medical records of 35 patients who had undergone the surgery for ANAET with a postoperative follow-up period of 6 months or more were reviewed. The main outcome measures were postoperative esodeviation angle, final success rate, and factors affecting surgical outcome. Surgical success was considered to be an alignment within 8 prism diopters (PD) at distance and near.

**Results:**

The preoperative mean esodeviation angles were 37.3 ± 13.7 PD at distance and 38.6 ± 16.6 PD at near. The postoperative mean esodeviation angles at distance were as follows: 4.2 PD at day 1, 4.0 PD at month 1, 3.9 PD at month 3, 4.9 PD at month 6, 4.7 PD at year 1, and 4.8 PD at final follow-up. There was no statistically significant difference in angle of esodeviation between the initial postoperative period (day 1 to month 6) and the final follow-up day (*p* > 0.05). The surgical success rate at final follow-up was 65.7% (23/35). Among the 12 patients for whom the surgery failed, 9 (24.3%) showed esotropia and 3 (8.1%) exotropia of more than 8 PD. Six patients (16.2%) underwent reoperation (4 for esotropia and 2 for exotropia). There was no factor influencing surgical outcome (*p* > 0.05).

**Conclusions:**

The surgical outcome in patients with ANAET was relatively favorable: the final success rate was 65.7% and the reoperation rate was 17.1%.

## Background

Acquired nonaccommodative esotropia (ANAET) is a type of strabismus characterized by a constant nonaccommodative esodeviation that develops after 6 months of age, in the absence of any significant refractive error and in an otherwise healthy child or adult [1, 2]. Many studies have identified factors affecting surgical outcome in infantile esotropia and accommodative esotropia. However, the literature regarding surgical outcomes or the factors influencing them in ANAET is thin, notwithstanding the research demonstrating older age at onset as a factor associated with postoperative stereopsis.

The purpose of the present study was to analyze the surgical outcomes in patients who had undergone surgery for ANAET and to determine the factors associated with favorable outcome.

## Methods

### Design and patients

We retrospectively reviewed the medical records of 35 patients who had undergone surgery for ANAET with a postoperative follow-up period of 6 months or more. ANAET was diagnosed if esotropia developed after 6 months of age in an otherwise healthy child and esodeviation angles at distance and near were unchanged even after the full correction of hyperopia of +2.0 diopters (D) or more, if revealed in cycloplegic refraction. Patients with any history of accommodative or partial accommodative esotropia, infantile esotropia, paralytic or restrictive strabismus, previous extraocular muscle surgery were excluded. And we included only the patients whose difference between the preoperative esodeviation angles at distance and near were less than 5 prism diopters (PD). Patients with associated findings of dissociated vertical deviation (DVD), latent nystagmus or inferior oblique overaction (IOOA) were excluded regardless of history, due to concern for unrecognized infantile esotropia. In the very young age group, we obtained the information about onset of strabismus which was acute, subacute, acquired or congenital from their parents or previous photography. This study was approved as a retrospective study by the Institutional Review Board of Hallym University Medical Center (IRB No. 2015-10-117) before data collection in order to review patient records and use the data, and adheres to the tenets of the Declaration of Helsinki. Informed written consent was obtained from all participants or their parents. 

We noted the following preoperative characteristics: sex, age at onset, age at surgery, refractive error, symptom duration, amblyopia, stereopsis, dominance of fixation, accompanying strabismus (e.g. vertical strabismus [≥ 5 PD in the primary position]), preoperative follow-up period, and type of surgery.

Cycloplegic refraction (with 1% cyclopentolate and 1% tropicamide) and fundus examination were performed on all of the patients. One operating surgeon measured all deviations using the alternate prism cover test at near and at distance (0.3 m and 6 m) (with spectacle correction based on cycloplegic refraction if necessary). Preoperative measurements were made no more than 3 days prior to surgery. The presence of amblyopia was defined as a difference of two or more lines between the best-corrected visual acuities of the right and left eyes or a best-corrected visual acuity lower than 20/30 on the Snellen visual acuity chart. The sensory status was evaluated by Titmus stereotest (Stereo Optical Co., Chicago, II, USA) at 33 cm and by Worth-4-dot test (Worth-4-dot Attachment; Richmond Products, Albuquerque, NM, USA) at 6 m.

### Surgical techniques

All of the surgeries were performed under general anesthesia by a single surgeon (D.G.C.). The selection of the surgical procedure was determined by the preoperative angle of esodeviation, the presence of dominance of fixation and the patient’s and surgeon’s preference. Fourteen patients underwent bilateral medial rectus recession (BMR), 17 unilateral medial rectus muscle recession and lateral rectus muscle resection (RR), and the remaining 4, with esotropia of 20 PD or less, unilateral medial rectus recession (UMR).

The extent of recession and resection was measured from the original muscle insertion. No hang-back or adjustable sutures were used. Surgical dosages were determined, based on the formulas suggested by Parks [3] and Wang [4] (Table 1).

**Table 1:** Surgical dosages for acquired nonaccommodative esotropia (ANAET)

Esodeviation (PD)	BMR (mm)	RR (mm)	UMR (mm)
15-19	3.5	3.5/4.5	5.5
20-24	4.0	4.0/5.5	6.0
25-29	4.5	4.5/6.5	
30-34	5.0	5.0/7.0	
35-39	5.5	5.5/7.5	
40-44	6.0	6.0/8.0	
45-49	6.5	6.5/8.0	
50-59	7.0	7.0/8.5	
60-69	7.5		

*BMR* bilateral medial rectus recession, *RR* unilateral medial rectus recession and lateral rectus resection, *UMR* unilateral medial rectus recession, *PD* prism diopters

### Main outcome measures

Postoperative alignment was measured on postoperative day 1, month 1, 3, 6, year 1, and at final follow-up. The main outcome measures included the postoperative esodeviation angles at each follow-up day, the final success rate, and the factors affecting surgical outcomes (i.e. sex, age at onset, age at surgery, refractive error, symptom duration, amblyopia, stereopsis, fusion by Worth-4-dot, alternate fixation, accompanying strabismus, preoperative follow-up period, and type of surgery). Surgical success was defined as esotropia or exotropia of 8 PD or less at distance and near.

### Statistics

SPSS software V.12.0 K (SPSS Inc., Chicago, Illinois, USA) was employed for the statistical analysis. The Wilcoxon signed rank test was used to compare the preoperative and postoperative angles of deviation. The Mann-Whitney U test and Fisher’s exact test were applied to analyze the demographic data. *P*-values less than 0.05 were considered significant.

## Results

The baseline characteristics of the total 35 patients are summarized in Table 2. The mean age at onset was 13.3 ± 17.5 years (range: 1.4-66.8 years), and the mean age at surgery was 20.3 ± 20.0 years (range: 2.5-72.2 years). The mean spherical equivalent was −0.7 ± 4.5, and the mean symptom duration was 4.3 ± 5.9 years. The mean preoperative follow-up period was 11.2 ± 29.5 months (range: 3-149 months), and the mean postoperative follow-up period was 57.8 ± 60.0 months (range: 6-201 months). Seven patients had amblyopia, which was defined as a two-line difference in acuity between eyes. For 10 patients, alternate fixation was possible. Five patients presented with vertical strabismus. The mean preoperative angle of esodeviation was 37.3 ± 13.7 PD (range: 15-75 PD) at distance and 38.6 ± 16.6 PD (range: 15-85 PD) at near.

**Table 2:** Demographic data on ANAET patients

Variables	*N* = 35
Sex (Male: Female)	20: 15
Age at onset (years)	13.3 ± 17.5 (range: 1.4 - 66.8)
Age at surgery (years)	20.3 ± 20.0 (range: 2.5 - 72.2)
Spherical equivalent	−0.7 ± 4.5 (range: −14.0 - +5.3)
Symptom duration (years)	4.3 ± 5.9 (range: 0.1 - 23.2)
Preoperative follow-up period (months)	11.2 ± 29.5 (range: 1 - 149)
Amblyopia	6 (17.1%)
Stereoacuity by Titmus test (seconds of arc)	(*n* = 22)
Stereopsis of 40-60	3 (13.6%)
Stereopsis of 80-3000	10 (45.5%)
No stereopsis	9 (40.9%)
Fusion by Worth-4-dot	3 / 23 (13.0%)
Alternate fixation	11 (31.4%)
Accompanying strabismus	
Vertical strabismus	5 (14.3%)
Preoperative angle of esodeviation (PD)	
Distance	37.3 ± 13.7 (range: 15 - 75)
Near	38.6 ± 16.6 (range: 15 - 85)
Type of surgery	
BMR	14
RR	17
UMR	4
Postoperative follow-up period (months)	57.8 ± 60.0 (range: 6-201)

*ANAET* acquired nonaccommodative esotropia, DVD dissociated vertical deviation, *PD* prism diopters, *BMR* bilateral medial rectus recession, *RR* unilateral medial rectus recession and lateral rectus resection, *UMR* unilateral medial rectus recession

### Surgical outcomes

The mean esodeviation angles at distance and at near were 4.2 and 3.8 PD at postoperative day 1 and 4.8 and 4.8 PD at final follow-up, respectively. There was no statistically significant difference in angle of esodeviation between the initial postoperative period (day 1 to month 6) and the final follow-up day (*p* > 0.05, Wilcoxon signed rank test, Tables 3 and 4). The initial postoperative angles of deviation were stably maintained during the follow-up period.

**Table 3:** Mean angle (PD) of esodeviation at distance

	Angle of esodeviation	*P*-value^a^
Preoperative	37.3 ± 13.7	0.000
Postoperative day 1	4.2 ± 8.4	0.772
Postoperative month 1	4.0 ± 5.3	0.751
Postoperative month 3	3.9 ± 7.0	0.432
Postoperative month 6	4.9 ± 7.0	0.497
Postoperative year 1	4.7 ± 6.4	0.916
Final follow-up	4.8 ± 15.1	

*PD* prism diopters

^a^Wilcoxon signed rank test (comparison with final follow-up day)

**Table 4:** Mean angle (PD) of esodeviation at near

	Angle of esodeviation	*P*-value^a^
Preoperative	38.6 ± 16.6	0.000
Postoperative day 1	3.8 ± 6.7	0.446
Postoperative month 1	4.2 ± 5.2	0.581
Postoperative month 3	4.6 ± 6.5	0.700
Postoperative month 6	4.1 ± 6.6	0.672
Postoperative year 1	5.5 ± 6.5	0.934
Final follow-up	4.8 ± 16.1	

*PD* prism diopters

^a^Wilcoxon signed rank test (comparison with final follow-up day)

The surgical success rates at postoperative day 1 were 88.6%: the final success rates were 65.7% (Table 5).

**Table 5:** Surgical success rates^a^ for ANAET

	Surgical success rates
Postoperative day 1	88.6%
Postoperative month 1	80.0%
Postoperative month 3	80.0%
Postoperative month 6	80.0%
Postoperative year 1	72.7%
Final follow-up	65.7%

*ANAET* acquired nonaccommodative esotropia

^a^Surgical success was defined as esotropia or exotropia of 8 PD or less at distance and near

### Reoperation

Six patients underwent reoperation, 4 for recurrent or residual esotropia and 2 for consecutive exotropia. The mean interval period between the 1^st^ and 2^nd^ operation was 71.8 ± 37.2 months (range: 25-120 months) (Table 6).

**Table 6:** Reoperation after surgery for ANAET

	*N* = 6
Reason for reoperation	
Recurrent or residual esotropia	4
Consecutive exotropia	2
Preoperative angle of esodeviation before 1^st^ operation (PD)	At distance: 40.0 ± 12.2 At near: 48.3 ± 17.6
Preoperative angle of esodeviation before 2^nd^ operation (PD)	At distance: 6.7 ± 29.8 At near: 10.8 ± 31.5
Mean interval period between 1^st^ and 2^nd^ operation (months)	71.8 ± 37.2 (range: 25 - 120)
Surgical success rate after reoperation	4/6 (66.6%)

*ANAET* acquired nonaccommodative esotropia, *PD* prism diopters

### Factors affecting surgical outcome

We analyzed the factors affecting surgical success (i.e. sex, age at onset, age at surgery, refractive error, symptom duration, amblyopia, stereopsis, fusion by Worth-4-dot, alternate fixation, accompanying strabismus [e.g. vertical strabismus], preoperative follow-up period, and type of surgery). There was no factor influencing surgical outcome (*P* > 0.05, Table 7).

**Table 7:** Patient characteristics in Success^a^ and Non-success ANAET Groups

Variable	Success Group (*n* = 26)	Non-success Group (*n* = 9)	*P*-value
Sex (Male: Female)	15: 11	5: 4	
Age at onset (years)	10.0 ± 12.1	19.3 ± 24.7	0.392^b^
Age at surgery (years)	18.8 ± 17.7	22.7 ± 23.9	0.674^b^
Spherical equivalent	−1.3 ± 5.6	0.5 ± 3.2	0.628^b^
Duration of symptom (years)	3.9 ± 4.7	5.0 ± 8.4	0.837^b^
Preoperative follow-up period (months)	12.1 ± 32.1	9.8 ± 25.9	0.420^b^
Amblyopia	5 (19.2%)	1 (11.1%)	0.891^c^
Stereopsis by Titmus test (seconds of arc)	(*n* = 16)	(*n* = 6)	
Stereopsis of 40-60	3 (18.8%)	0 (0%)	0.235^c^
Stereopsis of 80-3000	7 (43.8%)	3 (50.0%)	0.456^c^
No stereopsis	6 (37.5%)	3 (50.0%)	0.443^c^
Worth-4-dot	(*n* = 17)	(*n* = 6)	
Fusion	2 (11.8%)	1 (16.7%)	0.306^c^
Alternate fixation	5	0	0.374^c^
Accompanying strabismus			
Vertical strabismus	2	3	0.125^c^
Preoperative angle of esodeviation (PD)			
Distance	36.6 ± 10.3	38.5 ± 18.0	0.940^b^
Near	37.7 ± 13.9	40.5 ± 22.2	1.000^b^
Type of surgery			
BMR	11	3	0.581^c^
RR	13	4	0.285^c^
UMR	2	2	0.134^c^
Postoperative follow-up period (months)	45.8 ± 51.5	78.2 ± 68.3	0.203^b^

*ANAET* acquired nonaccommodative esotropia, *DVD* dissociated vertical deviation, *PD* prism diopters, *BMR* bilateral medial rectus recession, *RR* unilateral medial rectus recession and lateral rectus resection, *UMR* unilateral medial rectus recession

^a^Surgical success was defined as esotropia or exotropia of 8 PD or less at final follow-up(at both distance and near)

^b^Mann-Whitney U test; ^c^Fisher’s exact test

## Discussion

Conventionally, ANAET is considered to occur infrequently, but is sometimes associated with intracranial tumor or other central nervous system (CNS) lesions [5–14]. However, Jacobs et al. [1] reported that with this form of esotropia, neurologic problems are only rarely present. In this study we excluded any patient with known neurological disorder.

According to Jacobs et al. [1]’s report, approximately three-quarters of those who underwent surgery for ANAET had good alignment after a mean duration of 1 decade, and two-thirds of them were within 10 PD of orthotropia. Chan et al. [2] found that 64.7% of patients had successful outcomes after surgery for ANAET. Sturm et al. [15] reported a 92% surgical success rate (within 8PD or less of orthotropia) among acute acquired concomitant esotropia patients. Our results indicated the final success rates (an alignment within 8 PD) of 65.7%, which are in line with the findings of the relevant previous studies.

However, surgical failures for persistent esotropia were 3 times more numerous than for consecutive exotropia in this study. This would mean that the correction of the surgical tables might be considered. Because the study population was too small, the true difference between the groups could be hidden. Further prospective study involving more data would be needed.

In the present study, 6 patients (17.1%) underwent reoperation compared with 26.7 and 5.9% in the Jacobs et al. [1] and Chan et al. [2] investigations, respectively. These differences were thought to be owed to the significantly varying mean postoperative follow-up periods: 57.8 months (present study), 10.9 years (Jacobs et al.), and 17.8 months (Chan et al.).

Several studies about the surgical outcome of ANAET including Jacobs et al. [1] and Chan et al. [2] had the age limit of the childhood at onset or surgery. However, there was no limitation in the age of onset or surgery, so in this respect, there is some limitation in comparing the surgical result for ANAET between our study and the other studies mentions above. Because of this, we analyzed the success rates after separating the two groups by age, more than 18 years old or not. The surgical success rate of less than 18 years old group(1.4 ~ 17.5 years old, mean 10.8 years old) was 66.7% and that of more than 18 years old group(18.9 ~ 66.8 years old, mean 42.5 years old) was 54.5%. It was a little difference between the two groups. However, there was no statistical difference between two groups (*p* = 0.374, Fisher’s exact test).

The main limitation of our study is the non-standardized and retrospective data collection. The other limitation was that the sample was so diverse and included 6 patients with amblyopia and 3 with perfect stereopsis. And moreover, the range of refractive errors was from −14.0 to +5.3 D even though all of the patients were eligible for according to the diagnostic criteria of ANAET. Because the patients in whom esotropia had developed after 6 months of age were included in this study, even though patients with DVD, latent nystagmus or IOOA were excluded, the possibility infantile esotropia patients to be included in this study group were not completely ruled out. Further, a large prospective study looking at only individuals with childhood onset ANAET will be needed.

## Conclusions

In conclusion, this paper provides data on the clinical characteristics of, and surgical outcomes for, ANAET. The surgical outcome at final follow-up was favorable. And there was no factor influencing surgical outcome.

## Abbreviations

ANAET: Acquired nonaccommodative esotropia
BMR: Bilateral medial rectus recession
CNS: Central nervous system
DVD: Dissociated vertical deviation
IOOA: Inferior oblique overaction
IRB: Institutional Review Board
PD: Prism diopters
RR: Unilateral medial rectus muscle recession and lateral rectus muscle resection
UMR: Unilateral medial rectus recession

## References

[1] Jacobs SM, Green-Simms A, Diehl NN, Mohney BG (2011). Long-term follow-up of acquired nonaccommodative esotropia in a population-based cohort. Ophthalmology.

[2] Chan TY, Mao AJ, Piggott JR, Makar I (2012). Factors affecting postoperative stereopsis in acquired nonaccommodative esotropia. Can J Ophthalmol.

[3] Parks MM. Pediatric ophthalmology. 2^nd^ edition. Chapter 67: strabismus surgery. 1997. p. 976.

[4] Wang L, Wang X (2012). Comparison between graded unilateral and bilateral medial rectus recession for esotropia. Br J Ophthalmol.

[5] Von Noorden GF (1985). Esodeviations. Burian-von Noorden’s binocular vision and ocular motility: theory and management of strabismus.

[6] Astle WF, Miller SJ (1994). Acute comitant esotropia: a sign of intracranial disease. Can J Ophthalmol.

[7] Hoyt CS, Good WV (1995). Acute onset concomitant esotropia: when is it a sign of serious neurological disease?. Br J Ophthalmol.

[8] Macpherson H, De Becker I, MacNeill JR (1996). Beware: armed and dangerous-acquired non-accommodative esotropia. Am Orthopt J.

[9] Williams AS, Hoyt CS (1989). Acute comitant esotropia in children with brain tumors. Arch Ophthalmol.

[10] Greenberg AE, Mohney BG, Diehl NN, Burke JP (2007). Incidence and types of childhood esotropia: a population-based study. Opthalmology.

[11] Mohney BG (2001). Acquired nonaccommodative esotropia in childhood. J AAPOS..

[12] Simon JW, Waldman JB, Couture KC (1996). Cerebellar astrocytoma manifesting as isolated, comitant esotropia in childhood. Am J Ophthalmol.

[13] Harada T, Ohasi T, Ohki K, Sawamura Y, Yoshida K, Ito T (1997). Clival chordoma presenting as acute esotropia due to bilateral abducens palsy. Opthalmologica.

[14] Fukuo Y, Abe T, Hayasaka S (1998). Acute comitant esotropia in a boy with head trauma and convulsions receiving carbamazepine. Ophthalmologica.

[15] Sturm V, Menke MN, Knecht PB, Schoffler C (2011). Long-term follow-up of children with acute acquired concomitant esotropia. J AAPOS.

